# Federated learning algorithms for generalized mixed-effects model (GLMM) on horizontally partitioned data from distributed sources

**DOI:** 10.1186/s12911-022-02014-1

**Published:** 2022-10-16

**Authors:** Wentao Li, Jiayi Tong, Md. Monowar Anjum, Noman Mohammed, Yong Chen, Xiaoqian Jiang

**Affiliations:** 1 School of Biomedical Informatics, UTHealth, 7000 Fannin St, Houston, 77030 TX USA; 2 Department of Biostatistics, Epidemiology and Informatics, University of Pennsylvania, 3400 Civic Center Boulevard, Philadelphia, 19104 PA USA; 3 Department of Computer Science, University of Manitoba, Winnipeg, Canada

**Keywords:** GLMM, Federated learning, Mixed effects, Laplace approximation, Gauss–Hermite approximation

## Abstract

**Objectives:**

This paper developed federated solutions based on two approximation algorithms to achieve federated generalized linear mixed effect models (GLMM). The paper also proposed a solution for numerical errors and singularity issues. And showed the two proposed methods can perform well in revealing the significance of parameter in distributed datasets, comparing to a centralized GLMM algorithm from R package (‘lme4’) as the baseline model.

**Methods:**

The log-likelihood function of GLMM is approximated by two numerical methods (Laplace approximation and Gaussian Hermite approximation, abbreviated as LA and GH), which supports federated decomposition of GLMM to bring computation to data. To solve the numerical errors and singularity issues, the loss-less estimation of log-sum-exponential trick and the adaptive regularization strategy was used to tackle the problems caused by federated settings.

**Results:**

Our proposed method can handle GLMM to accommodate hierarchical data with multiple non-independent levels of observations in a federated setting. The experiment results demonstrate comparable (LA) and superior (GH) performances with simulated and real-world data.

**Conclusion:**

We modified and compared federated GLMMs with different approximations, which can support researchers in analyzing versatile biomedical data to accommodate mixed effects and address non-independence due to hierarchical structures (i.e., institutes, region, country, etc.).

## Introduction

### Background

There is an increasing surge of interest in analyzing biomedical data to improve health [1]. Biostatisticians and machine learning researchers are keen to access personal health information for a deeper understanding of diagnostics, disease development, and potential preventive or treatment options [2].

In the US, healthcare and clinical data are often collected by local institutions. For many situations, combining these datasets would increase statistical power in hypothesis testing and provide better means to investigate regional differences and subpopulation bias (e.g., due to differences in disease prevalence or social determinants). However, such an information harmonization process needs to respect the privacy of individuals, as healthcare data contain sensitive information about personal characteristics and health conditions. As a minimum requirement [3], HIPAA (Health Insurance Portability and Accountability Act) [4] specifies PHIs (protected health information) and regulations to de-identify the sensitive information (i.e., safe harbor mechanism). But HIPAA compliance does not mean full protection of the data, as several studies demonstrated re-identifiability of HIPAA de-identified data [5–7]. Ethical healthcare data sharing and analysis should also respect the “minimum necessary” principle to reduce the unnecessary risk of potential data leakage, which might increase the likelihood of information leakage.

**Fig. 1:**
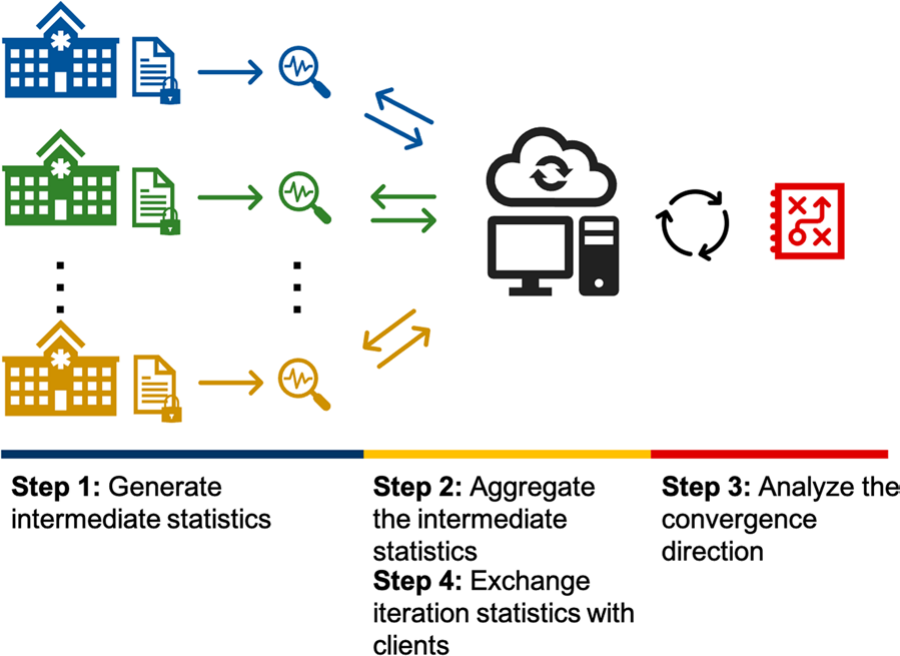
Schema of federated learning model in multiple geographically distributed healthcare institutions. The local institutions periodically exchange intermediate statistics and update the convergence situation of the global model

The recent development of federated learning, which intends to build a shared global model without moving local data from their host institutions (Fig. 1), shows good promise in addressing the challenge in data sharing mentioned above. Despite the exciting progress [8–11], there is still an important limitation as existing models cannot effectively handle mixed-effects (i.e., both fixed and random effects), which is very important to analyzing non-independent, multilevel/hierarchical, longitudinal, or correlated data [12]. Also, due to the sampling errors (i.e., smaller sample size in local sites), variances from these local statistics are larger than those of the global model. These issues, if not addressed appropriately, would lead to failure in global optimization. The goal of this paper is to improve existing techniques and provide practical solutions with open-source implementation and to allow ordinary biomedical/healthcare researchers to build federated mixed effect learning models for their studies.

### Related work

Federated learning for healthcare data analysis is not a new topic, and there have been many previous studies in biomedical field. For example, predicting outcomes from distributed clinical notes and Electronic Health Record (EHR) data [13, 14], federated Natural Language Processing models [15], Internet of medical Things [16], and many predictive machine learning models [17–19]. However, many of the existing methods assume the observations are independent and identically distributed, such as GLORE [20] and FL-QSAR [21]. In the presence of non-independence due to hierarchical structures (e.g., due to institutional or regional differences), existing federated models have strong limitations in ignoring the regional differences. The generalized linear mixed model (GLMM), which takes the heterogeneous factors into consideration, is more amenable to accommodate the heterogeneity across healthcare systems. There have been very few studies in this area and one relevant work is a privacy-preserving Bayesian GLMM model [22], which proposed an Expectation-Maximization (EM) algorithm to fit the model collaboratively on horizontally partitioned data. The convergence process is relatively slow (due to the Metropolis-Hastings sampling in the E-step) and it is also not very stable (likely to be trapped in local optima [23] in high-dimensional data). In the experiment, a loose threshold (i.e., 0.08) was used as a convergence condition [22] while typical federated learning algorithms [20] in healthcare use much stringent convergence threshold (i.e., $10^{-6}$).

Another related work to fit GLMM in a federated manner is the distributed penalized quasi-likelihood (dPQL) algorithm [24]. This algorithm reduces the computational complexity by considering the target function of penalized quasi-likelihood, which is motivated from Laplacian approximation. The model has communication efficiency over the EM approach and can converge in a few shots. However, the target function PQL can have first order asymptotic bias [25] due to the Laplacian approximation (LA) of the integrated likelihood.

There is an alternative strategy, Gauss–Hermite (GH), which supports high-order approximation. It is computationally more intensive and requires special techniques to handle the numerical instability of the logSumExp operation (due to the overflow issue when the dimensionality grows in the sum of the exponential terms). The difference between GH and LA is that GH approximates the model using a higher degree of polynomials, which makes GH more accurate but less efficient. But, when the number of federated nodes increases, the LA can accomplish the same task more than three-times faster.

In this paper, we proposed new approaches to support federated GLMM with LA and GH approximation. We also addressed the optimization challenges with new methods due to the federated computation and compare their performance on simulated and real-world data to demonstrate the practicability of our proposed models.

## Methods

In this section, we will discuss the statistic model along with challenges to be tackled. A high-level schema of the method is shown in algorithm 1.

### Notation

Before we introduce the formation of GLMM, let us define some notations.

**Table:** 

*i*	Index of sites	$l_i$	Log-likelihood function for site *i*
*j*	Index of patients in a specific site	${\beta }$	Parameters of fixed effect
*k*	Index of Hermite polynomial	$\mu _i$	Parameters of random effect in site *i*
*K*	Order of Hermite polynomial	$\tau$	Hyper-parameters
*m*	Number of sites	${\theta }$	Parameter space $({\beta },\tau )$
$n_i$	Number of patients in site *i*	$X_{ij}$	A vector represents the data of *j*-th patient in *i*-th site
${\mathcal {L}}_i$	Likelihood function for site *i*	$y_{ij}$	The outcome of patient *j* from site *i*
$\lambda$	The parameter of regularization term	*p*	Number of variables

### Fitting GLMM with quasi-likelihood

Let us provide the formation of the GLMM. Define ${\mathbb {P}}$ is the distribution of interest and depending on patient-level data $X_{ij},\ y_{ij}$. Define $\phi$ as the distribution of random effects. We can compose the joint distribution as following

$$\begin{aligned} \prod _{j=1}^{n_i}{\mathbb {P}}(\varvec{\theta }|X_{ij},y_{ij})\phi (\mu _{i};\tau ) \end{aligned}$$

Now we have the log-likelihood function of the joint distribution:

1 $$\begin{aligned} \log \{{\mathcal {L}}(\varvec{\theta })\}=\sum _{i=1}^{m}\log \left\{ \int _{\mu _{i}} \left[ \prod _{j=1}^{n_i}{\mathbb {P}}(\varvec{\theta }|X_{ij},y_{ij})\right] \phi (\mu _{i};\tau )\text {d}\mu _{i}\right\} \end{aligned}$$

From the log-likelihood function Eq. (1), one can see that it does not support direct linear decomposition. In order to support federated learning, we will leverage approximation strategies to make the objective linearly decomposable with simple summary statistics.

We will compare Laplace approximation and Gauss–Hermite approximation in the following sections.

### Laplace (LA) approximation

With the help of Laplace approximation, the integration from Eq. (1) can be approximated by an exponential family expression.

2 $$\begin{aligned} \int _{\mu _{i}}f_{\theta }(\mu _{i})\text {d}\mu _{i}=\int _{\mu _{i}}e^{\log {f_ {\varvec{\theta }}(\mu _{i})}}\text {d}\mu _{i}\triangleq \int _{\mu _{i}}e^{g(\mu _{i},\theta )}\text {d}\mu _{i} \end{aligned}$$

After the deduction in Additional file 1: Appendix proofs A.1, the intractable problem is solved and the objective is to maximize the following formula with respect to $\theta$, where *g* is an exponential family function defined above (Eq.(2))

$$\begin{aligned} \sum _{i=1}^{n_i}\left( g({\hat{\mu }}_{i},\varvec{\theta }) -\dfrac{n_i}{2}\log \left( g_{\mu \mu }({\hat{\mu }}_{i},\varvec{\theta })\right) )\right) \end{aligned}$$,

for which the terms are linearly decomposable from local sites. Site *i* needs to calculate the following aggregated data:

- $$p\times p$$ matrix:
  3 $$\begin{aligned} \dfrac{{\hat{\omega }}_{\beta \beta }{\hat{\omega }}-{\hat{\omega _\beta {\hat{\omega }}}}_ {\beta }}{{\hat{\omega }}^2}+{\hat{\mu }}_{\beta \beta }g_\mu +{\hat{\mu _\beta }} ({\hat{\mu }}_{\beta }g_{\mu \mu }+g_{\mu \beta })+{\hat{\mu }}_{\beta }g_{\mu \beta }+g_{\beta \beta } \end{aligned}$$
- *p* - dim vector:
  4 $$\begin{aligned} \dfrac{{\hat{\omega _\beta }}}{{\hat{\omega }}}+{\hat{\omega }}^2g_{\mu \beta }({\hat{\mu _i}})g_\mu +g_\beta \end{aligned}$$
- scalar of random effect: ${\hat{\mu _i}}$ and first order derivative of $\tau$ by
  $$\begin{aligned} \dfrac{{\hat{\omega _\tau }}}{{\hat{\omega }}}+{\hat{\omega }}^2g_{\mu \tau }({\hat{\mu _i}})g_\mu +g_\tau \end{aligned}$$
  where $${\hat{\omega }}=\sqrt{-\dfrac{1}{g_{\mu \mu }({\hat{\mu }}_{i0})}}$$

### Gauss–Hermite (GH) approximation

Gauss–Hermite approximation [26] implements Hermite interpolation concerning Eq. (2). And after the deduction in Additional file 1: Appendix proofs A.2, notice that when the order of Hermite polynomial $K=1$, the objective function is identical to the method with Laplace approximation. Because GH is more generalizable, we will describe the distributed federated learning model on the GLMM problem with the formation of Gauss–Hermite approximation in Additional file 1: Appendix proofs A.2 Eq. (2). For each site *i*, the followings need to calculate and transmit:

- $$p\times p$$ matrix:
  5 $$\begin{aligned}&\dfrac{{\hat{\omega }}_{\beta \beta }{\hat{\omega }}-{\hat{\omega _\beta {\hat{\omega }}}}_ {\beta }}{{\hat{\omega }}^2}+\dfrac{1}{\sum _{k=1}^Kf_k}\sum _{k=1}^K\dfrac{\partial }{\partial \varvec{\beta }}(f_{k_\mu }{\hat{\mu _\beta }}+f_{k_\omega }{\hat{\omega _\beta }}+f_{k_\beta }) \nonumber \\ -&\dfrac{1}{(\sum _{k=1}^Kf_k)^2}\Vert \sum _{k=1}^l(f_{k_\mu }{\hat{\mu _\beta }}+f_{k_\omega }{\hat{\omega _\beta }}+f_{k_\beta })\Vert ^2_2 \end{aligned}$$
- *p* - dim vector:
  6 $$\begin{aligned} \dfrac{{\hat{\omega _\beta }}}{{\hat{\omega }}}+\dfrac{1}{\sum _{k=1}^Kf_k}\sum _{k=1} ^K(f_{k_\mu }{\hat{\mu _\beta }}+f_{k_\omega }{\hat{\omega _\beta }}+f_{k_\beta }) \end{aligned}$$
- scalar of random effect: ${\hat{\mu _i}}$ and first order derivative of $\tau$ by
  $$\begin{aligned} \dfrac{{\hat{\omega _\tau }}}{{\hat{\omega }}}+\dfrac{1}{\sum _{k=1}^Kf_k}\sum _{k=1}^K(f_{k_\mu } {\hat{\mu _\tau }}+f_{k_\omega }{\hat{\omega _\tau }}+f_{k_\tau }) \end{aligned}$$

### Training Penalization GLMM with GH approximation

The convergence of the approximation of the likelihood function may be compromised due to over-fitting. Also, for those spatially correlated data, the convergence of them may lead to a complex model. Hence, *L*2 regularization is added to the local log-likelihood function of Gauss–Hermite approximation form, and as shown below

7 $$\begin{aligned} l_i=\log {\mathcal {L}}_i=\log \left( \sqrt{2\pi }{\hat{\omega \sum }}_{k=1}^Kh_k \exp \left\{ g({\hat{\mu }}_{i}+\sqrt{2\pi }{\hat{\omega }} x_k;\varvec{\theta })+x_k^2\right\} \right) - \lambda \Vert \varvec{\beta }\Vert ^2_2 \end{aligned}$$

note that when $K=1$, it is represented as regularized Laplace approximation to the problem. To evaluate and find the optimum $\lambda$, we steadily increased the value of $\lambda$ in range [0, 10] by 1. Set $\lambda _{\text {opt}}$ as the optimized regularization term with largest $\sum _i^ml_i$. And choose $\hat{\varvec{\beta }}_{\text {opt}}$ as the optimized estimator for $\varvec{\beta }$.

Due to the limited computation digits, computers are not able to calculate the correct results of the local log-likelihood function $l_i$ of the Gauss–Hermite approximation form as stated above. Such problem is also known as the Log-Sum-Exponential problem and can be solved by shifting the center of the exponential sum for easier computation,

$$\begin{aligned} \log \sum _{k=1}^K\exp \left\{ g({\hat{\mu }}_{i}+\sqrt{2\pi }{\hat{\omega }} x_k;\varvec{\theta })+x_k^2\right\} =a+\log \sum _{k=1}^K\exp \left\{ g({\hat{\mu }}_{i}+\sqrt{2\pi }{\hat{\omega }} x_k;\varvec{\theta })+x_k^2-a\right\} \end{aligned}$$

where *a* is an arbitrary number.

Thus, the global problem of maximizing $\sum _i^ml_i$ can be divided into several local maximization problems Eq. (7). Each local site *i* will update the regression intermediates, and they will be combined to update the iteration status. Specifically, in each iteration of the federated GLMM algorithm, the following statistics are exchanged from each site to contribute aggregated data through Federated Averaging (FedAvg) for the global model

**Table:** 

LA	GH
Number of variables *p*	Number of variables *p*
$p\times p$ matrix (Eq. (3))	$p\times p$ matrix (Eq. (5))
*p* - dim vector (Eq. (4))	*p* - dim vector (Eq. (6))
*p* - dim vector ${\beta }$	*p* - dim vector ${\beta }$
Scalar $\lambda$	Scalar $\lambda$
Scalar ${\hat{\mu _i}}$	Scalar ${\hat{\mu _i}}$
Scalar first order derivative $\tau$	Scalar first order derivative $\tau$
	Scalar *K*

Detailed derivatives with the logistic regression setting of the optimization are presented in the Additional file 1: Appendix proofs A.3.
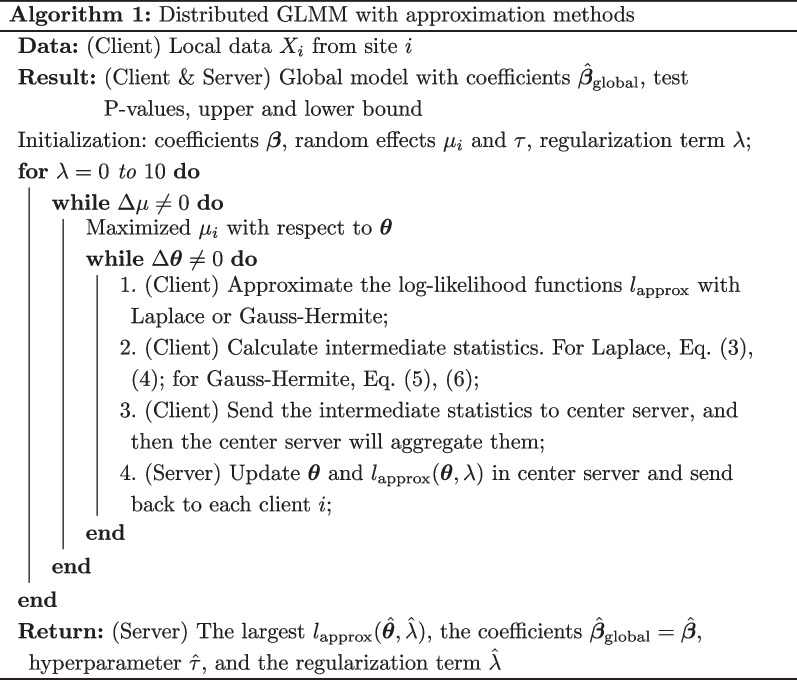


## Results

Our algorithm is developed in Python with packages *pandas*, *numpy*, *scipy*, and the benchmark algorithm is glmer function in R package ‘lme4’.

### Benchmarking the methods using synthetic data

To test the performance of our proposed methods, we first designed a stress test based on a group of synthetic data, which include 8 different settings (Table 1), and each set contains 20 datasets. In each dataset, it consists of 4 categorical variables with value in $\{0,1\}$; 6 categorical variables with value in range $[-1,1.5]\in {\mathbb {R}}$; 1 outcome variable with value in $\{0,1\}$; Site ID, represents the id of which site the entry belongs to; Site sample size, represents the number of samples in this specific setting; Log-odds ratio for each sample; Number of true positive, true negative, true positive, false positive, false negative.

To evaluate which method can reach better performance, we proposed the following evaluation measurements: discrimination of the estimated coefficients $\hat{\varvec{\beta }}$, the test power of each coefficient, and the precision and recall of the number of significant coefficients.

**Table 1:** The summary of data in each setting

Setting	Number of sites	Sample size in eachsite	Variance
1	2	500	Small
2	2	500	Large
3	10	500	Small
4	10	500	Large
5	2	30	Small
6	2	30	Large
7	10	30	Small
8	10	30	Large

The valuation experiments were conducted among federated GLMM with Laplace approximation, federated GLMM with Gauss–Hermite approximation, and centralized GLMM (all of the data stored in single host) in the R package. And the stress test will be run in 160 different datasets in 8 different settings as mentioned in Table 1. All of the data in different settings were randomly separated into training sets and validation sets with a ratio of 7:3. And we trained the federated learning model on training data sets, then by slowly increasing the regularization term $\lambda$, we chose the optimum model with the best Akaike information criterion and Bayesian information criterion performance on the validation sets. All testing was performed on 2017 iMac with 16 GB memory, CPU (4.2 GHz Quad-Core Intel Core i7), macOS Big Sur version 11.6, Python 3.8, and R version 3.5.0.

**Fig. 2:**
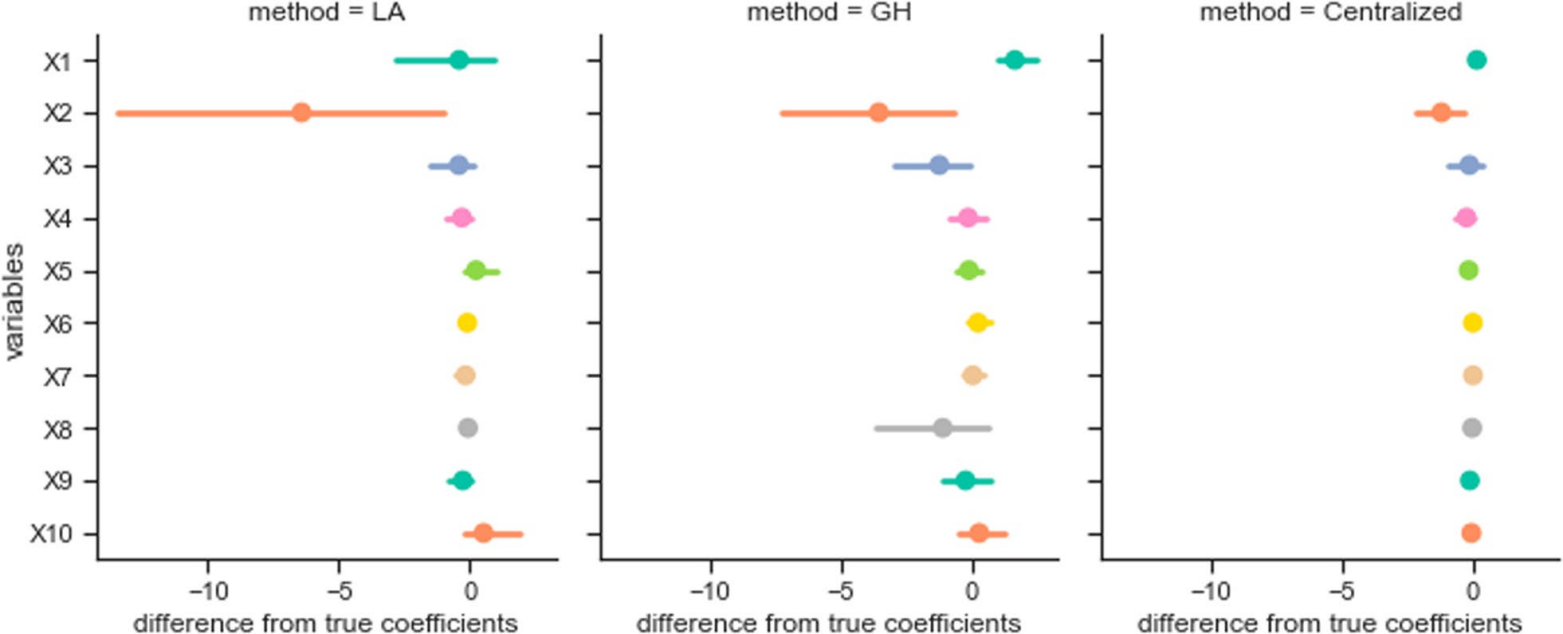
The difference from coefficients to the true parameters that are used to generate data. (Left) The distributed GLMM with Laplace approximation; (Middle) The distributed GLMM with 2-degree Gauss–Hermite approximation. Reminds that $X_1$ is the intercept; (Right) The benchmark of centralized GLMM in R package

Although we tested the data sets with the state-of-art benchmark algorithm for centralized GLMM in R, the regression is not perfect for the ground truth coefficients we used to generate the data (Fig. 2). So, it is also important to have the *p* values of variables into consideration when interpreting the model. Thus, We made comparisons among centralized GLM, centralized GLMM, federated Laplace (LA) method, and federated Gauss–Hermite (GH) method concerning the *p* values of coefficients. Additional file 1: Tables 1, 2, 3 in the Appendix B captured the performance of different methods.

**Fig. 3:**
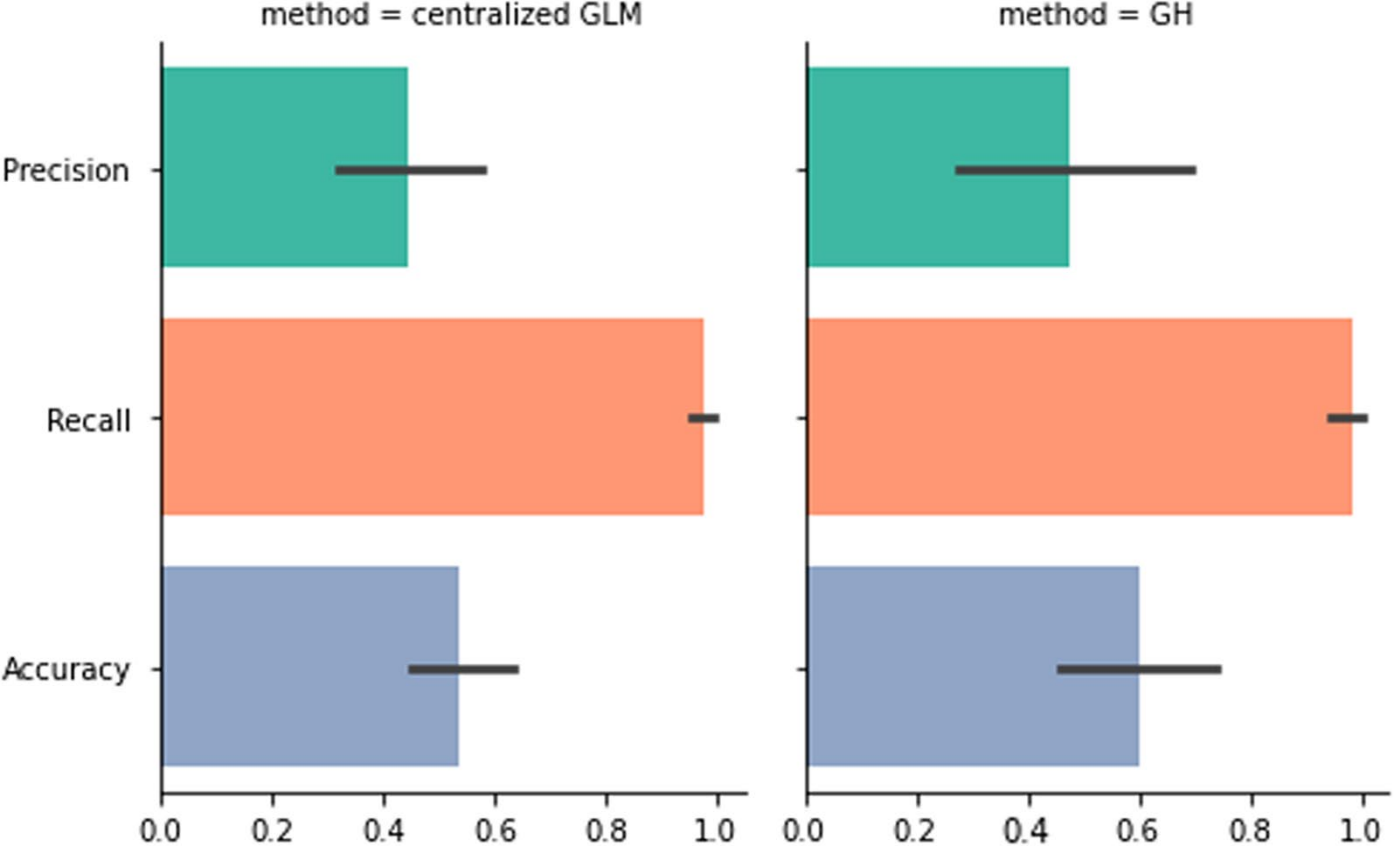
The comparison of performances between centralized GLM and federated GH models. (Left) The centralized GLM with Logistic Regression; (Right) The federated GLMM with 2-degree GH approximation. All methods are applied to all 8 settings of synthetic data that pooled together. Method “centralized GLM”is done with R packages “lm” in a centralized settings

**Fig. 4:**
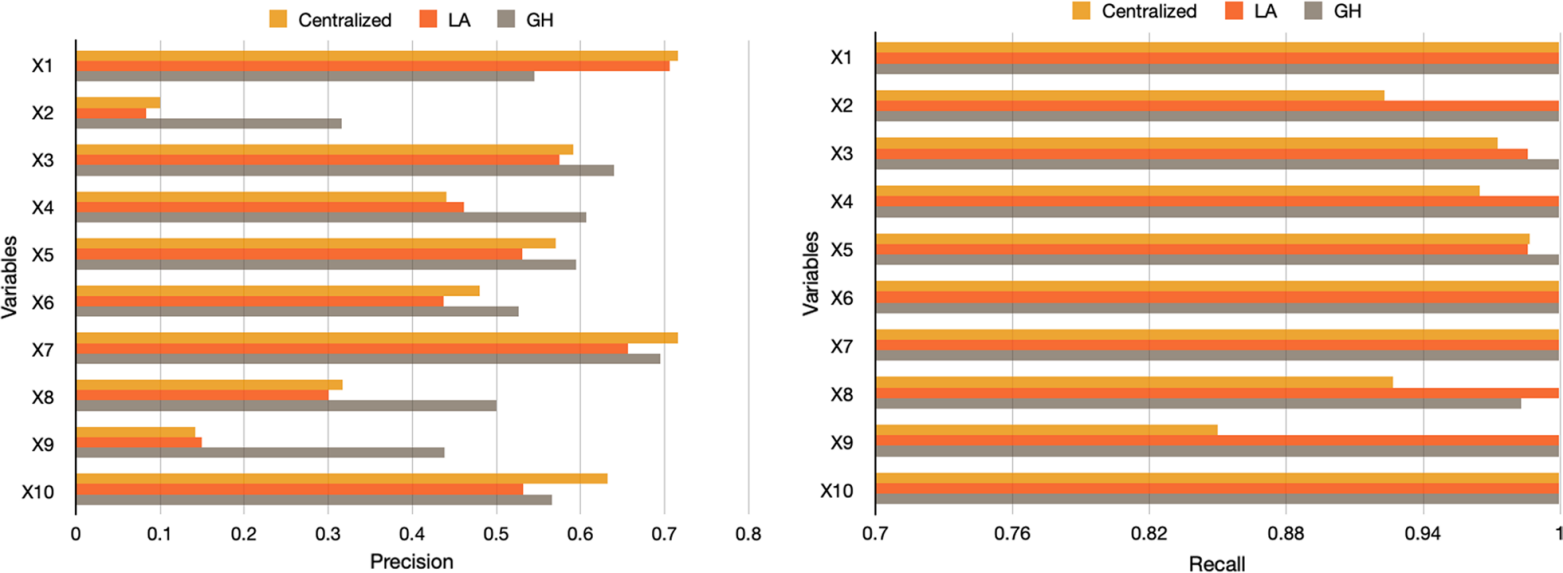
The precision and recall in variable-wise among centralized, Laplace, and Gauss–Hermite method under significance level $\alpha =0.05$. (Left) The precision of the test compared to the true value. (Right) The recall of the test compared to the true value

First, we show the increase of performance using mixed effect models in heterogeneous data. Figure 3 shows we can train models with mixed-effects estimation (centralized GLMM and federated GH) to outperform the model with fixed-effects-only estimation (GLM). Because GLMM can handle the site-wise bias by estimating random-effects, GLMM-based methods performed better than GLM method in federated settings (local data generation/collection process naturally includes random effects. Figure 4 shows the precision and recall results in variables-wise of centralized, Laplace, and Gauss–Hermite methods. Noted that we set our Gauss–Hermite approximation to 2-degree, thus GH method can achieve higher accuracy and better performance in estimating the significance of variables due to the higher-degree approximation.

**Fig. 5:**
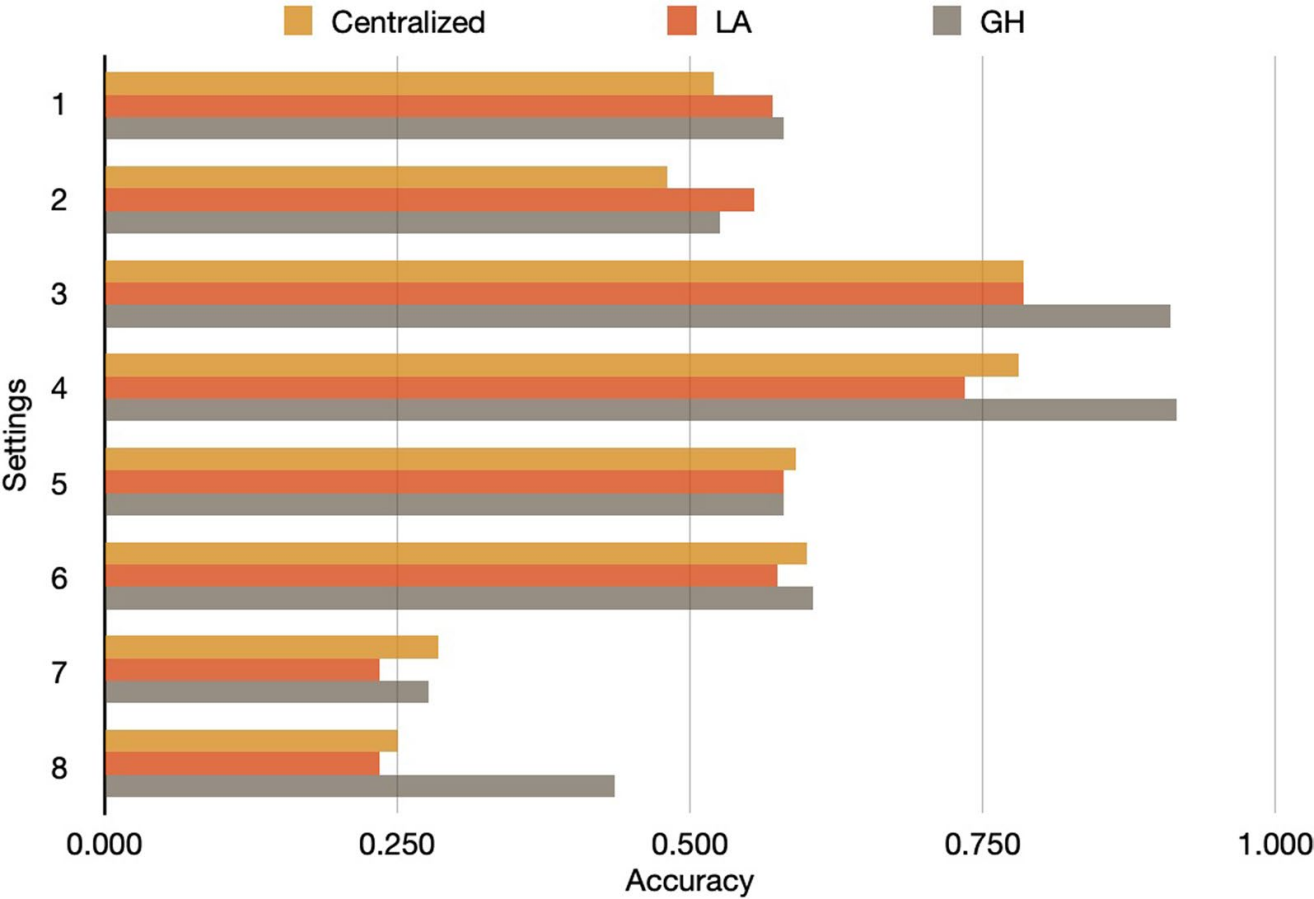
The accuracy in 8 different data settings among centralized, Laplace, and Gauss–Hermite method under significance level $\alpha =0.05$. The performance of models in different number of nodes (2 vs 10), sample size (500 vs 30), and variance (large vs small)

**Fig. 6:**
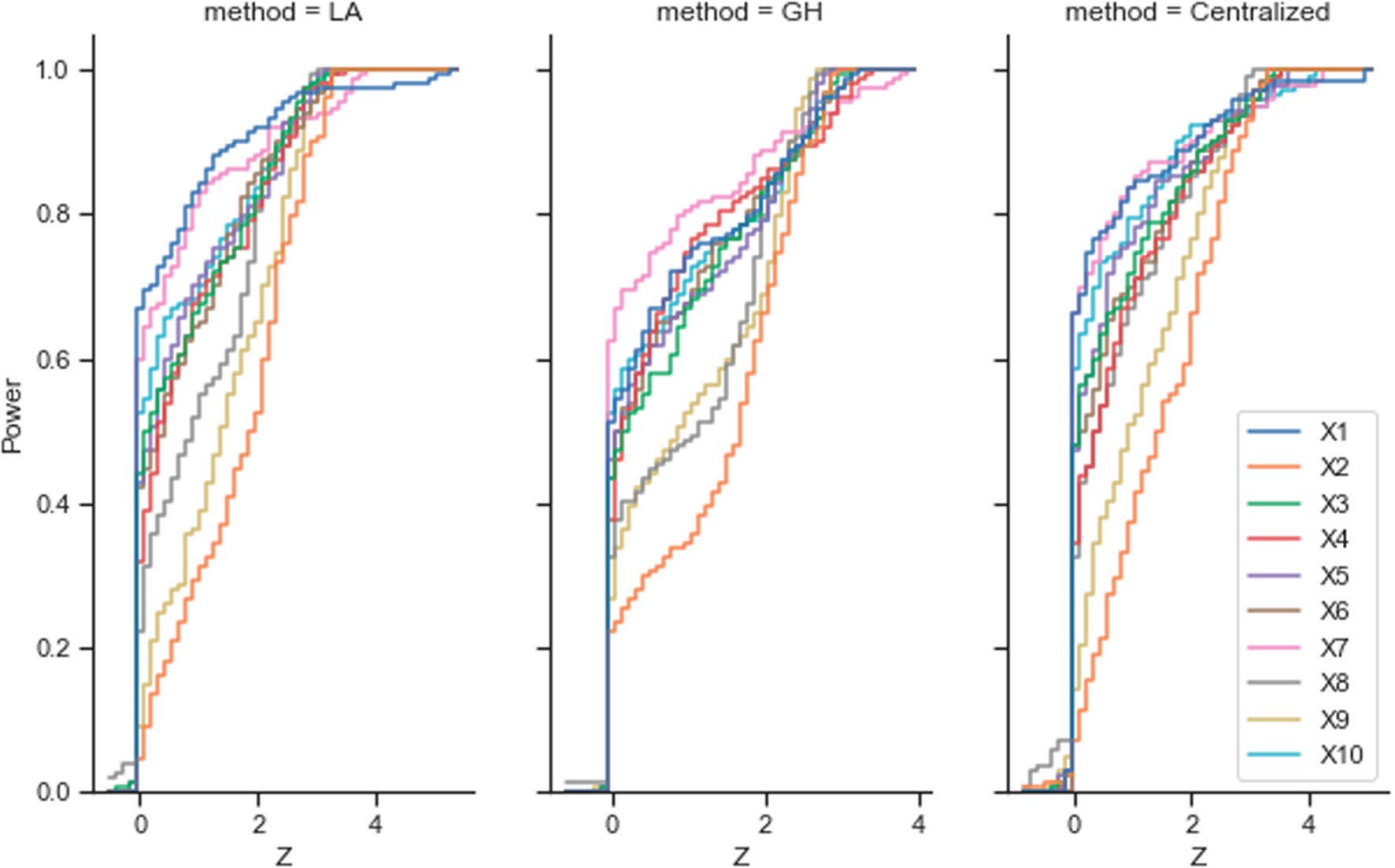
The curve of test power among centralized, Laplace, and Gauss–Hermite methods. (Left) The power of the test of the Laplace method. (Middle) The power of the test of the 2-degree Gauss–Hermite method. (Right) The power of the test of the Centralized method. Power was calculated as the two-sided t-test on *p* values among different methods

**Table 2:** The convergence rates on approximation methods LA and GH. (Both LA and GH held the same convergence threshold $10^{-3}$. The mean values and standard deviations (in parentheses) were given)

Setting	LA		GH	
	Steps	Runtime (s)	Steps	Runtime (s)
1	22.875 (21.623)	47.953 (20.513)	34.850 (9.213)	104.460 (10.614)
2	21.500 (21.977)	40.947 (36.466)	35.000 (8.711)	100.940 (19.940)
3	29.867 (31.719)	108.931 (65.486)	34.900 (6.138)	1259.285 (231.956)
4	27.846 (24.034)	84.343 (76.502)	36.650 (6.310)	1342.695 (250.603)
5	59.722 (42.057)	10.631 (3.945)	33.750 (10.146)	12.568 (2.116)
6	67.188 (48.994)	10.499 (4.054)	31.400 (11.081)	11.430 (3.064)
7	96.286 (53.635)	96.501 (38.632)	37.450 (3.818)	369.165 (41.998)
8	116.083 (46.479)	91.304 (62.410)	37.150 (4.295)	309.693 (36.621)

The simulation results showed the federated Gauss–Hermite approximation performed better than the method based on Laplace approximation on every variable. Also, the federated Gauss–Hermite method achieved higher test power (Fig. 6). From the result (Fig. 5), we can see that the federated GH method outperformed the Centralized LA and the federated LA methods, thanks to its better approximation using higher-degree functions. Here, we use the true parameters and their *p* values set during the data the synthetic data generation process as the golden standard. The accuracy means the alignment ratio of three different methods against the gold standard. As illustrated by the table and figure below, we compare the results (Settings 1, 2 vs. Settings 3, 4). One can tell that when the number of nodes/sites increases, the performance in estimating the significance of parameters becomes better because of the increase in total sample size (1000 total samples in Settings 1, 2 and 5000 total samples in Settings 3, 4). If the sample size in each node/site is too small, considering the comparison pair (Settings 5, 6 vs Settings 7, 8), the increases in the number of nodes/sites will largely decrease the performance of all three models. Because the estimation of the random effects in such a small sample size is limited, adding more nodes/sites to the federated network will decrease the performance of the fixed effect parameter significance estimation. We also measured the impact of heterogeneity on these methods considering random effects. Seeing the pairs (Setting 1 vs. Setting 2), (Setting 3 vs. Setting 4), (Setting 5 vs. Setting 6), and (Setting 7 vs. Setting 8), the heterogeneity of nodes/sites did not affect the three models obviously. Since the Centralized GLMM, federated LA, and federated GH methods all consider the random effects in the model, they can decently handle the impact of data heterogeneity across nodes/sites. This is not the case for generalized linear model (GLM), which does not consider the random effects across sites . When considering the convergence rates between the two approximation methods, both showed less convergence efficiency in Setting 7 and 8 (Table 2). The result Indicates that more local sites and smaller sample sizes will make the federated GLMM more inefficient to converge. Also, GH approximation method will required more computation time compared with LA approximation. In sum, one-degree increase of the approximation function in LA with our developed GH method, GH outperformed LA methods for federated GLMM implementation.

### Mixed-effects logistic regression on mortality for patients with COVID-19

We analyzed the data of COVID-19 electronic health records collected by Optum$^\circledR$ from February 2020 to January 28, 2021, from a network of healthcare providers. The dataset has been de-identified and based on HIPAA statistical de-identification rules and managed by Optum$^\circledR$ customer data user agreement. In this database, there are 56,898 unique positive tested COVID-19 patients. After removing the patients with missing data, the final cohort contains 4,531 patients who died and the rest population (41,781) survived. The database contains a regional variable with five levels (Midwest, Northwest, South, West, Others/unknown) to provide privacy-preserving area information to indicate where the samples were collected.

We have conducted a GLMM model (considering region-distinct random effect) using this dataset with the following predictors: age, gender, race, ethnicity, Chronic obstructive pulmonary disease (COPD), Congestive heart failure (CHF), Chronic kidney disease (CKD), Multiple sclerosis (MS), Rheumatoid arthritis (RA), LU (other lung diseases), High blood pressure (HTN), ischemic heart disease (IHD), diabetes (DIAB), Asthma (ASTH), obesity (Obese). Our proposed method with GH approximation performed the best with both the smallest Akaike information criterion (AIC) and Bayesian information criterion (BIC) according to the table of the goodness of fit (Table 3). And the performance of different methods can be shown in (Table 4).

**Table 3:** Statistics of goodness of fit among different methods

	Log-likelihood	AIC	BIC
R	13562.9	27165.9	27340.8
LA	− 13695.0	27428.0	27594.1
GH	− 11.8	61.6	227.7

**Fig. 7:**
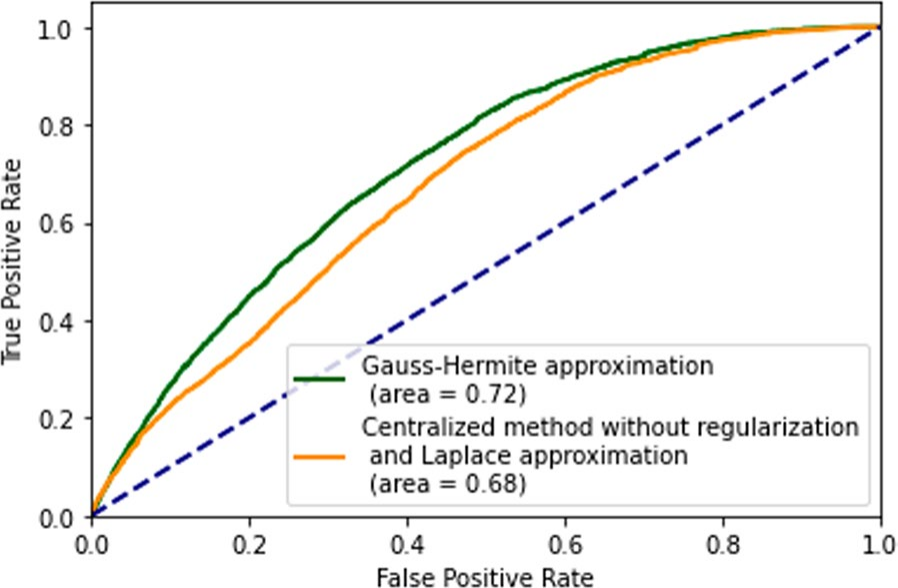
The ROC curve with Area Under Curve (AUC) among centralized, Laplace, and Gauss–Hermite methods. The orange ROC curve is the centralized method without regularization and the Laplace approximation(i.e., R implementation in the ‘lme4’ package, which does not have an option for including regularization). AUC values are also included, a higher AUC value implicates better performance of the model. The green ROC curve is the 2-degree Gauss–Hermite method with regularization

**Table 4:** Statistics of performances among different methods (95% CIs were generated by Wilson Score interval)

		Precision	Recall	F1-score	AUC	threshold
Centralized method with LA	Value	0.1507	0.6204	0.2425	0.6789	0.0900
	Lower bound (0.95)	0.1474	0.6160	0.2386	0.6700	
	Upper bound (0.95)	0.1539	0.6248	0.2464	0.6878	
GH with regularization	Value	0.1705	0.6546	0.2705	0.7178	0.0108
	Lower bound (0.95)	0.1670	0.6503	0.2664	0.7091	
	Upper bound (0.95)	0.1739	0.6589	0.2745	0.7265	

We also compared the ROC curves (Fig. 7) between our proposed GH method and centralized method to check their performance. And the result showed that GH approximation (AUC=0.72) outperforms the centralized method without regularization (AUC=0.68). Indicating GH-based GLMM method has better classification performance than the GLMM based on LA approximation. In our proposed model, it showed variables: Unknown race, Chronic kidney disease (CKD), Multiple sclerosis (MS), and other lung diseases (LU) are not significant to the mortality of COVID-19. The result of the regression is in the Additional file 1: Appendix B (Tables 4, 5, 6).

## Conclusion

In this paper, we developed solutions to address the limited digit problem (i.e., overflow issue of fixed-length object types due to extremely large numbers in local estimation) using an alternative loss-less estimation of log-sum-exponential term, and the singularity issue (involved in Newton optimization) with an adaptive regularization strategy to avoid inverting low-rank matrices without imposing too much unnecessary smoothness.

We further compared two federated GLMM algorithms with our developed federated solutions (LA vs. GH) and demonstrated the performance of the federated GLMM based on the GH method surpassed the method based on LA in terms of the accuracy of estimation, power of tests, and AUC. Although the GH method is requiring slightly more computations than the LA method, it is still acceptable for more accurate results. For example, in the prediction of COVID-19 mortality rates, the accuracy of prediction will be more reliable, as we have shown in the previous section.

## Discussion

Notice there is a trade-off between the accuracy and scalability of federated learning models. On the one hand, the GH model has high accuracy because of the better approximation through high-order statistics. On the other hand, deploying heavier models can be difficult and time-consuming, in these cases, a simpler linear approximation like LA has more advantages. It is important to consider these factors when selecting a model for federated learning.

So, for those federated learning tasks that require high-accuracy *p* values estimation on parameters and have a relatively small group of training nodes, the GH method is considered better than the LA method. For example, cross-silo genes association test within several cohorts. This example study aims to determine the risk genes from logistic regression models on genetic and phenotype data. Since such an example study is accuracy-sensitive to the significance of target genes and gene data repositories are often at a smaller scale, the GH method will suit well under this scenario.

On the other hand, the LA method should fit better in those federated learning tasks with a large number of training nodes and high-efficiency requirements. For example, the risk factors analysis of worldwide pandemic disease (i.e., COVID-19). Since the outbreak of worldwide pandemic is normally rapid and vast, it is crucial to gather as much information as possible in a short time and to take appropriate actions. By adopting our proposed LA method, one can deploy a privacy-preserving factor analysis model with high efficiency.

During the optimization iterations, we noticed that some sites have already achieved convergence in very few steps. If those sites stop communicating with the central server, they can be released from extra computations. We would investigate more efficient algorithms based on such a strategy of ‘lazy regression’ for minimizing communication for federated learning models. Also, we will include different federated aggregation strategies to our future works. Since sending intermediate information can not protect the training process intact (i.e. poison attack by malicious user with designed information sent to the aggregator), further investigation and implementation in secure multi-party computation technique like SecAgg [27] and LightSecAgg [28] is our next step.

Another limitation of the proposed federated GLMM model is not yet differentially private and iterative summary statistics exchange can lead to incremental information disclosure, which might increase the re-identification risk over time. There are several strategies to improve the model based on secure operations like homomorphic encryption and differential privacy, which we have previously studied in GLM models [29]. Finally, in practice, there can be extra heterogeneity that cannot be explained by random intercepts only, it is of interest to further develop our algorithms toward GLMM that allows multiple random effects including random coefficients in the regression models.

## Supplementary Information

**Additional file 1.** The supplementary file contains two parts. Part A contains detailed proofs with respect to Laplace approximationand Gauss Hermite approximation. And the derivatives of federated learning with Gauss Hermite approximation;Part B contains tables of Performance in precision, recall, true-negative rate, and accuracy rate. Each table hasdetailed explanation on a specific experiment.

## Data Availability

The synthetic datasets with generated code during the current study are available in the GitHub repository, https://github.com/Li-Wentao/Simulation_data_GLMM.git. The data that support the findings of COVID-19 are available from Optum$^\circledR$ but restrictions apply to the availability of these data, which were used under license for the current study, and so are not publicly available.

## Abbreviations

LA: Laplace approximation
GH: Gauss–Hermite approximation
