# Supplementary material for: Federated learning algorithms for generalized mixed-effects model (GLMM) on horizontally partitioned data from distributed sources
Source: BMC Med Inform Decis Mak. 2022 Oct 16;22:269. doi: 10.1186/s12911-022-02014-1 (PMC9569919; doi:10.1186/s12911-022-02014-1)
Supplement: Supplementary file 1 — Additional file 1. The supplementary file contains two parts. Part A contains detailed proofs with respect to Laplace approximationand Gauss Hermite approximation. And the derivatives of federated learning with Gauss Hermite approximation;Part B contains tables of Performance in precision, recall, true-negative rate, and accuracy rate. Each table hasdetailed explanation on a specific experiment. [file 12911_2022_2014_MOESM1_ESM.pdf]

Appendices

A Supplementary proofs

A.1 Laplace approximation

Let us explain the Laplace approximation. Denote that

$$f_\theta(\mu_i) \triangleq \prod_{j=1}^{n_i} \mathbb{P}(\theta|X_{ij}, y_{ij})\phi(\mu_i;\tau)$$

and one can see the log term inside the log-likelihood function that

$$\int_{\mu_i} f_\theta(\mu_i)\mathrm{d}\mu_i = \int_{\mu_i} e^{\log f_\theta(\mu_i)}\mathrm{d}\mu_i \triangleq \int_{\mu_i} e^{g(\mu_i,\theta)}\mathrm{d}\mu_i \tag{1}$$

Applying a Taylor expansion on $g(\mu_i, \theta)$, and we choose $\hat{\mu}_i$ that maximized $g(\mu_i, \theta)$. See that $\hat{\mu}_i$ satisfies $g_\mu(\hat{\mu}_i, \theta) = 0$ and $g_{\mu\mu}(\hat{\mu}_i, \theta) < 0$, we have

$$g(\mu_i, \theta) = g(\hat{\mu}_i, \theta) - \frac{1}{2}(\hat{\mu}_i - \mu_i)^2 \left(-g_{\mu\mu}(\hat{\mu}_i, \theta)\right) + o(\mu_i^2)$$

With Laplace approximation, main text Eq.(2) can approximate as

$$\int_{\mu_i} e^{g(\mu_i,\theta)}\mathrm{d}\mu_i \approx exp\{g(\hat{\mu}_i, \theta)\} \left[2\pi \cdot -\frac{1}{g_{\mu\mu}(\hat{\mu}_i, \theta)}\right]^{n_i/2}$$

A.2 Gauss Hermite approximation

The Hermite polynomial $H_k(x)$ and weight h_k are defined as followings,

$$H_k(x) \triangleq (-1)^k e^{x^2} \frac{d^k}{dx^k} e^{-x^2} \qquad h_k \triangleq \frac{2^{k-1}k!\sqrt{\pi}}{k^2 \left[H_{k-1}(x_k)\right]^2}$$

where x_k are the roots of $H_k(x) = 0$.

Thus, with the Gauss-Hermite approximation, main text Eq.(2) can be approximated by

$$\int_{\mu_i} e^{g(\mu_i)}\mathrm{d}\mu_i \approx \sqrt{2\pi}\hat{\omega} \sum_{k=1}^{K} h_k \exp\left\{g(\hat{\mu}_i + \sqrt{2\pi}\hat{\omega}x_k) + x_k^2\right\}, \quad \hat{\omega} = \sqrt{-\frac{1}{g''(\hat{\mu}_i)}} \tag{2}$$

notice that when $K = 1$, it is a Laplace approximation.

Our final objective function for GH is

$$\mathcal{L}_i = \mathcal{L}_i(\boldsymbol{\beta}, \mu_i; X_{i\cdot}, y_{i\cdot}) = \sqrt{2\pi}\hat{\omega} \sum_{k=1}^{K} h_k \exp\left\{g(\hat{\mu}_i + \sqrt{2\pi}\hat{\omega}x_k; \boldsymbol{\beta}) + x_k^2\right\}$$

Denote that

$$\begin{aligned}
f_k &= h_k \exp\left\{g(\hat{\mu}_i + \sqrt{2\pi}\hat{\omega}x_k; \boldsymbol{\beta}) + x_k^2\right\} \\
f_{k_\beta} &= f_k g_\beta(\hat{\mu}_i) \\
f_{k_\mu} &= f_k g_\mu(\hat{\mu}_i) \\
f_{k_\omega} &= f_k g_\mu(\hat{\mu}_i)\sqrt{2\pi}x_k
\end{aligned}$$

A.3 Optimization

A logistic regression model with random effects is developed under the form of

$$\log\{\mathcal{L}(\theta)\} = \sum_{i=1}^{m} \log\left\{\int_{\mu_i} \left[\prod_{j=1}^{n_i} \mathbb{P}(\theta|X_{ij}, y_{ij})\right] \phi(\mu_i; \tau)\mathrm{d}\mu_i\right\}$$

and the distribution $\mathbb{P}$ follows density of logit and ϕ is a univariate normal, see that

$$\prod_{j=1}^{n_i} \mathbb{P}(\theta|X_{ij}, y_{ij}) = \prod_{j=1}^{n_i} \pi_{ij}^{y_{ij}}(1-\pi_{ij})^{(1-y_{ij})}$$

$$\phi(\mu_i; \theta) = \frac{1}{\sqrt{2\pi}\tau} \exp(-\mu_i^2/2\tau^2)$$

where π_{ij} is a Sigmoid function of μ_i and defined as

$$\pi_{ij} = \frac{\exp(X_{ij}^\top \boldsymbol{\beta} + \mu_i)}{1 + \exp(X_{ij}^\top \boldsymbol{\beta} + \mu_i)}$$

with the Gauss-Hermite approximation set up, the objective function can be approximated as

$$\sqrt{2\pi}\hat{\omega} \sum_{k=1}^{K} h_k \exp\left\{g(\hat{\mu}_i + \sqrt{2\pi}\hat{\omega}x_k; \boldsymbol{\beta}) + x_k^2\right\}$$

where

$$\begin{aligned}
g(\mu_i; \boldsymbol{\beta}) &= \log \prod_{j=1}^{n_i} \mathbb{P}(\theta|X_{ij}, y_{ij})\phi(\mu_i; \tau) \\
&= \sum_{j=1}^{n_i} [\log \mathbb{P}(\theta|X_{ij}, y_{ij})] + \log \phi(\mu_i; \tau) \\
&= \sum_{j=1}^{n_i} [y_{ij} \log \pi_{ij} + (1-y_{ij}) \log(1-\pi_{ij})] + \log \phi(\mu_i; \tau)
\end{aligned}$$

A.3.1 Step 1: Maximize $g(\mu_i)$

To maximize $g(\mu_i)$, we need to get the derivatives

$$\begin{aligned}\frac{\partial g}{\partial \mu_i} &= \sum_{j=1}^{n_i}\left[y_{ij}\frac{1}{\pi_{ij}}\frac{\partial \pi_{ij}}{\partial \mu_i} - (1-y_{ij})\frac{1}{1-\pi_{ij}}\frac{\partial \pi_{ij}}{\partial \mu_i}\right] + \frac{1}{\phi}\frac{\partial \phi}{\partial \mu_i} \\ &= \sum_{j=1}^{n_i}(y_{ij}-\pi_{ij}) - \frac{\mu_i}{\tau^2}\end{aligned}$$

where

$$\frac{\partial \phi}{\partial \mu_i} = (\sqrt{2\pi}\tau)^{-1}\exp(-\mu_i^2/2\tau^2)\cdot(-\mu_i/\tau^2)$$

and

$$\frac{\partial^2 g}{\partial \mu_i^2} = -\sum_{j=1}^{n_i}\frac{\partial \pi_{ij}}{\partial \mu_i} - \frac{1}{\tau^2} < 0$$

where

$$\frac{\partial \pi_{ij}}{\partial \mu_i} = \frac{\exp\left(X_{ij}^\top \boldsymbol{\beta} + \mu_i\right)}{\left[1+\exp\left(X_{ij}^\top \boldsymbol{\beta} + \mu_i\right)\right]^2}$$

see that it is a convex problem, using newton's method, we can derive $\hat{\mu}_i = \arg\max_{\mu_i} g(\mu_i)$ that is global optimum.

A.3.2 Step 2: Maximization preparation of β in LOCAL

See the derivative

$$\frac{\partial \pi_{ij}}{\partial \boldsymbol{\beta}} = \frac{X_{ij}\exp\left(X_{ij}^\top \boldsymbol{\beta} + \mu_i\right)}{\left[1+\exp\left(X_{ij}^\top \boldsymbol{\beta} + \mu_i\right)\right]^2}$$

and denote $f_k(\hat{\mu}_i;\boldsymbol{\beta}) := h_k \exp\left\{g(\hat{\mu}_i + \sqrt{2\pi}\hat{\omega}x_k;\boldsymbol{\beta}) + x_k^2\right\}$, then

$$\begin{aligned}\frac{\partial \mathcal{L}_i}{\partial \boldsymbol{\beta}} &= \sqrt{2\pi}\hat{\omega}\sum_{k=1}^{K}\left\{f_k(\hat{\mu}_i;\boldsymbol{\beta})h_k \left.\frac{\partial g(\mu_i;\boldsymbol{\beta})}{\partial \boldsymbol{\beta}}\right|_{\mu_i=\hat{\mu}_i+\sqrt{2\pi}\hat{\omega}x_k}\right\} && (3)\\ &= \sqrt{2\pi}\hat{\omega}\sum_{k=1}^{K}\left\{f_k(\hat{\mu}_i;\boldsymbol{\beta})h_k\sum_{j=1}^{n_i}(X_{ij}y_{ij} - X_{ij}\pi_{ij})\right\} && (4)\end{aligned}$$

and the second derivative

$$\begin{aligned}\frac{\partial^2 \mathcal{L}_i}{\partial \boldsymbol{\beta}^2} = \sqrt{2\pi}\hat{\omega}\sum_{k=1}^{K}\Bigg\{ & f_k(\hat{\mu}_i;\boldsymbol{\beta})h_k^2\sum_{j=1}^{n_i}(X_{ij}y_{ij} - X_{ij}\pi_{ij})\left[\sum_{j=1}^{n_i}(X_{ij}y_{ij} - X_{ij}\pi_{ij})\right]^\top && (5)\\ & + f_k(\hat{\mu}_i;\boldsymbol{\beta})h_k\sum_{j=1}^{n_i}\left(-X_{ij}\frac{\partial \pi_{ij}}{\partial \boldsymbol{\beta}}\right)\Bigg\} && (6)\end{aligned}$$

Notice that μ_i in (3), (4) and (5) are replaced by $\hat{\mu}_i + \sqrt{2\pi}\hat{\omega}x_k$ where $\hat{\mu}_i$ is the maximand of function $g(\cdot)$ with respect to μ_i.

A.3.3 Step 3: Maximization of β in GLOBAL

Reminds that $\mathcal{L} = \sum_{i=1}^m \log \mathcal{L}_i$, then another Newton's method is applied in global log-likelihood function,

$$\frac{\partial \mathcal{L}}{\partial \boldsymbol{\beta}} = \sum_{i=1}^{m} \frac{\mathcal{L}_i'(\boldsymbol{\beta})}{\mathcal{L}_i(\boldsymbol{\beta})} \qquad \frac{\partial^2 \mathcal{L}}{\partial \boldsymbol{\beta}^2} = \sum_{i=1}^{m} \left[\frac{\mathcal{L}_i''(\boldsymbol{\beta})}{\mathcal{L}_i(\boldsymbol{\beta})} - \left(\frac{\mathcal{L}_i'(\boldsymbol{\beta})}{\mathcal{L}_i(\boldsymbol{\beta})} \right)^2 \right]$$

Now, focus on $\boldsymbol{\beta}^{(n+1)} = \boldsymbol{\beta}^{(n)} - \dfrac{\mathcal{L}'(\boldsymbol{\beta}^{(n)})}{\mathcal{L}''(\boldsymbol{\beta}^{(n)})}$, deduce that

$$\frac{\mathcal{L}'(\boldsymbol{\beta}^{(n)})}{\mathcal{L}''(\boldsymbol{\beta}^{(n)})} = \frac{\sum_{i=1}^m \frac{\mathcal{L}_i'(\boldsymbol{\beta})}{\mathcal{L}_i(\boldsymbol{\beta})}}{\sum_{i=1}^m \frac{\mathcal{L}_i''(\boldsymbol{\beta})}{\mathcal{L}_i(\boldsymbol{\beta})} - \sum_{i=1}^m \left(\frac{\mathcal{L}_i'(\boldsymbol{\beta})}{\mathcal{L}_i(\boldsymbol{\beta})} \right)^2}$$

A.4 Synthetic data generation

There are 8 settings of data sets generated from the process, and each setting can be summarized in main text Tab.1. We set true sensitivity and specificity as $sen = 0.6$ and $sp = 0.9$ and

$$\boldsymbol{\beta} = (-1.5, 0.1, -0.5, -0.3, 0.4, -0.2, -0.25, 0.35, -0.1, 0.5)$$

Also define $X_1 = \mathbb{1}_N$ as the intercept, and X_2, X_3, X_4 are generated with Bernoulli distribution with probability $p = 0.1, 0.3, 0.5$ respectively.

$$f(X_i; p) = \begin{cases} p & \text{if } X_i = 1 \\ q = 1 - p & \text{if } X_i = 0 \end{cases} \tag{7}$$

then X_5, X_6, X_7 are generated from normal distributions $\mathcal{N}(0, 0.5), \mathcal{N}(0, 1), \mathcal{N}(0, 1.5)$ respectively. Lastly, X_8, X_9, X_{10} are generate from uniform distributions $\mathcal{U}(-0.5, 0.5), \mathcal{U}(-0.7, 0.7)$, $\mathcal{U}(-1, 1)$ respectively. We also generate the random effect μ using trivariate normal distribution

$$\mathcal{N}_3 \left(\begin{pmatrix} 0 \\ 0 \\ 0 \end{pmatrix}, \Sigma \right), \quad \Sigma = I_3 \tag{8}$$

and with the settings, we can deduce the log-odds ratio with following formula

$$\log(\pi) = f(X\boldsymbol{\beta} + \mu + \epsilon) \tag{9}$$

where f is the sigmoid function defined as

$$f(x) = \frac{e^x}{1 + e^x} \tag{10}$$

Now, we generate the outcomes y for each sample with Bernoulli distribution where the log-odds ratio served as the probability p. Also, the sensitivity and specificity can be calculated with Binomial distribution with probability $sen + \mu_2$ and $sp + \mu_3$.

B Supplementary tables

Supplementary Table 1. The performance of centralized GLMM on R package with a significance threshold $\alpha = 0.05$. TNR refers to the True negative rate.

	**Precision**	**Recall**	**TNR**	**Accuracy**
X1	0.7162	1.0000	0.0000	0.7162
X2	0.1000	0.9231	0.2000	0.2635
X3	0.5917	0.9726	0.3467	0.6554
X4	0.4400	0.9649	0.2308	0.5135
X5	0.5703	0.9865	0.2568	0.6216
X6	0.4797	1.0000	0.0000	0.4797
X7	0.7162	1.0000	0.0000	0.7162
X8	0.3167	0.9268	0.2336	0.4257
X9	0.1417	0.8500	0.1953	0.2838

Supplementary Table 2. The performance of distributed GLMM with Laplace transformation with a significance threshold $\alpha = 0.05$. TNR refers to the True negative rate.

	**Precision**	**Recall**	**TNR**	**Accuracy**
X1	0.7063	1.0000	0.0000	0.7063
X2	0.0833	1.0000	0.2667	0.3125
X3	0.5750	0.9857	0.4333	0.6750
X4	0.4609	1.0000	0.3168	0.5688
X5	0.5303	0.9859	0.3034	0.6063
X6	0.4375	1.0000	0.0000	0.4375
X7	0.6563	1.0000	0.0000	0.6563
X8	0.3000	1.0000	0.3226	0.4750
X9	0.1500	1.0000	0.2817	0.3625
X10	0.5316	1.0000	0.0263	0.5375

Supplementary Table 3. The performance of distributed GLMM with 2-degree Gauss-Hermite transformation with significance threshold $\alpha = 0.05$. TNR refers to True negative rate.

	**Precision**	**Recall**	**TNR**	**Accuracy**
X1	0.5455	1.0000	0.0000	0.5455
X2	0.3158	1.0000	0.3390	0.4935
X3	0.6404	1.0000	0.4938	0.7338
X4	0.6066	1.0000	0.4000	0.6883
X5	0.5952	1.0000	0.3544	0.6688
X6	0.5260	1.0000	0.0000	0.5260
X7	0.6948	1.0000	0.0000	0.6948
X8	0.5000	0.9828	0.4063	0.6234
X9	0.4386	1.0000	0.3846	0.5844
X10	0.5658	1.0000	0.0294	0.5714

Supplementary Table 4. The result of centralized GLMM in R package

	**Coef**	**Std.Err**	**z**	**P-value**	**[0.025**	**0.975]**
(Intercept)	-5.882	0.133	-44.294	0.000	-6.142	-5.621
age	0.043	0.001	30.918	0.000	0.040	0.045
Gen_M	0.370	0.033	11.052	0.000	0.304	0.435
race_Asian	0.365	0.122	2.995	0.003	0.126	0.604
race_Caucasian	0.157	0.049	3.185	0.001	0.061	0.254
race_Other.Unknown	0.385	0.070	5.530	0.000	0.249	0.521
ethnicity_Not.Hispanic	-0.181	0.060	-3.030	0.002	-0.299	-0.064
ethnicity_Unknown	-0.088	0.069	-1.274	0.203	-0.224	0.048
COPD_Y	0.096	0.038	2.556	0.011	0.022	0.169
CHF_Y	0.153	0.040	3.796	0.000	0.074	0.232
CKD_Y	0.029	0.044	0.674	0.500	-0.056	0.115
MS_Y	-0.090	0.124	-0.725	0.469	-0.332	0.153
RA_Y	0.144	0.070	2.048	0.041	0.006	0.281
LU_Y	-0.001	0.204	-0.004	0.997	-0.400	0.398
HTN_Y	0.113	0.053	2.121	0.034	0.009	0.217
IHD_Y	0.334	0.037	9.077	0.000	0.262	0.406
DIAB_Y	0.149	0.035	4.214	0.000	0.080	0.218
ASTH_Y	-0.170	0.054	-3.143	0.002	-0.276	-0.064
Obese_Y	0.211	0.043	4.893	0.000	0.126	0.295

Supplementary Table 5. The result of federated GLMM with GH method

	Coef	Std.Err	z	P-value	[0.025	0.975]
(Intercept)	-3.476	0.066	-53.064	0.000	-3.605	-3.348
age	0.043	0.001	55.794	0.000	0.041	0.044
Gen_M	0.370	0.021	17.847	0.000	0.329	0.410
race_Asian	0.364	0.075	4.847	0.000	0.217	0.511
race_Caucasian	0.156	0.028	5.538	0.000	0.101	0.211
race_Other.Unknown	0.383	0.041	9.365	0.000	0.303	0.463
ethnicity_Not.Hispanic	-0.178	0.036	-4.936	0.000	-0.249	-0.107
ethnicity_Unknown	-0.091	0.042	-2.154	0.031	-0.174	-0.008
COPD_Y	0.096	0.026	3.700	0.000	0.045	0.146
CHF_Y	0.154	0.029	5.205	0.000	0.096	0.211
CKD_Y	0.028	0.031	0.901	0.367	-0.033	0.090
MS_Y	-0.093	0.078	-1.205	0.228	-0.245	0.059
RA_Y	0.144	0.049	2.959	0.003	0.048	0.239
LU_Y	0.001	0.119	0.008	0.994	-0.233	0.235
HTN_Y	0.113	0.028	3.988	0.000	0.057	0.169
IHD_Y	0.334	0.024	14.162	0.000	0.288	0.381
DIAB_Y	0.149	0.022	6.677	0.000	0.105	0.192
ASTH_Y	-0.169	0.032	-5.242	0.000	-0.233	-0.106
Obese_Y	0.211	0.028	7.483	0.000	0.156	0.267

Supplementary Table 6. The result of federated GLMM with LA method

	Coef	Std.Err	z	P-value	[0.025	0.975]
(Intercept)	-3.162	0.064	-49.437	0.000	-3.288	-3.037
age	0.041	0.001	54.895	0.000	0.040	0.043
Gen_M	0.359	0.021	17.374	0.000	0.318	0.399
race_Asian	0.315	0.075	4.214	0.000	0.168	0.461
race_Caucasian	0.130	0.028	4.675	0.000	0.076	0.185
race_Other.Unknown	0.330	0.041	8.138	0.000	0.251	0.410
ethnicity_Not.Hispanic	-0.211	0.036	-5.886	0.000	-0.281	-0.140
ethnicity_Unknown	-0.114	0.042	-2.707	0.007	-0.197	-0.031
COPD_Y	0.098	0.026	3.750	0.000	0.047	0.149
CHF_Y	0.156	0.030	5.234	0.000	0.098	0.215
CKD_Y	0.029	0.032	0.928	0.353	-0.033	0.092
MS_Y	-0.092	0.077	-1.192	0.233	-0.244	0.059
RA_Y	0.138	0.049	2.829	0.005	0.042	0.234
LU_Y	-0.007	0.118	-0.061	0.952	-0.239	0.225
HTN_Y	0.104	0.028	3.722	0.000	0.049	0.159
IHD_Y	0.337	0.024	14.208	0.000	0.290	0.383
DIAB_Y	0.144	0.022	6.461	0.000	0.100	0.187
ASTH_Y	-0.175	0.032	-5.445	0.000	-0.238	-0.112
Obese_Y	0.182	0.028	6.450	0.000	0.127	0.237
